# Hospital Expenditure: The Commissariat

**Published:** 1896-03-28

**Authors:** 


### THE INSTITUTIONAL WORKSHOP.

### HOSPITAL EXPENDITURE.—THE COMMISSARIAT.

### XIII.—THE COST OF THE DIETS.

The average cost of the patients' diet, so far as it can be ascertained, has been shown to vary between 5s. and 6s. 6d. a week in certain London hospitals, and between 4s. and 6s. a week in certain provincial hospitals. Wider variations probably exist among the numerous institutions which are entirely unable to specify the sums expended in boarding this part of the household. For the mere effort to ascertain with accuracy how money is spent almost invariably results in curtailing superfluous items, and drawing those institutions in which it is the rule towards a common level of expenditure.

The food of the patients is one of the few points in hospital administration about which no mystery is maintained. Its quality is freely open in nearly all institutions to inspection, and the quantity allowed is prescribed on printed diet tables, which anyone may see. Were these adhered to the difference in cost between the patients' diet at one hospital and at another should not be difficult to estimate. But experience has shown that no classification of diets, however complete, can meet the requirements of an ever-varying stream of patients, and hence some variation in kind and quantity must be allowed for in many cases. Inquiry has shown that in numerous instances in which a rigid allowance of bread and other articles is prescribed in the diet table, no real restriction is placed on the appetite of the patient. The allowance simply represents the average quantity found sufficient, but anyone may have more if he please. And that this, for convalescent patients at any rate, is the more reasonable system few will dispute.

Taking the diet tables then to represent an average rate of consumption it becomes at once clear that the board of patients on low or fever diet is a less important item than that of the others, and that when the cost of the very light fare they are able to consume is deducted, a correspondingly larger proportion is available for the provisions of the remainder. A patient on milk diet alone will receive three pints of milk a day, cost 3¾d.; but ordinary low diet includes a good deal more. A few examples will be sufficient. Bread is reckoned at 1d. per pound. Beef tea at 4d. per pint (1 pound of meat to 1 pint); broth at 2d. a pint; milk at 10d. a gallon, which is slightly more than the average price paid.

Low diet, London Hospital: Bread, 10 oz., ¾d.; milk, 2 pints, 2½d.; broth, 1 pint, 2d.; cost, 5¼d. a day. Low diet, Middlesex: Bread, 10 oz., ¾d.; milk, 1 pint, 1¼d.; beef tea, ½ pint, 2d.; broth, 1 pint, 2d.; cost, 6d. a day. Low diet, Westminster: Bread, 8 oz., ½d.; milk, 1½ pints, 2d.; beef tea (9 oz. to the pint), 1 pint, 2¼d.; tea and sugar, ½d.; cost, 5¼d. Milk pudding is commonly added, and may cost an additional ¾d. a head.

Again, in provincial hospitals we find for low diet—Birmingham General: Bread, 12 oz., ¾d.; milk, 3 pints, 3¾d.; pudding, ¾d.; cost, 5¼d. a day. Bristol General: Bread, *ad lib.*, ¾d.; milk, 3 pints, 3¾d.; beef tea, 1 pint, 4d.; cost, 8½d. a day.

These diets are subject, of course, to modification, according to the nature of the case. The beef tea, though indicated in some tables and not in others, is practically ordered as an extra or omitted as the patient's needs suggest, and hence the variations shown in different tables are more apparent than real. The beef tea is the expensive item, and its cost is difficult to estimate accurately, since, as will be subsequently shown, the cost of its preparation varies enormously from one institution to another. Jelly or eggs are sometimes ordered in place of it, and any of these three may raise the cost of low diet to as much as 8d. a day. The usual average cost is from 4d. to 6d.

The proportion of patients in different hospitals on low diet varies very much according to the locality and the class of cases most frequently admitted. In the General Infirmary, Leeds, the average of patients on low diet for the year is given at 10 33 out of a daily average of 363 occupied beds, or about 3 per cent. of all the patients. At the Glasgow Western Infirmary the average number on milk diet is between 8 and 9 per cent. At Bradford Infirmary on a given day the number on simple diet was 64 out of 182, or 35 per cent. At the Newcastle Royal Infirmary the average for the last three years of those on low diet has been estimated at about 27 per cent., but apparently this is meant to include also those on poultry and fish, who numbered on a given day as many as 62 out of 70. If this is the usual proportion, the number on what is ordinarily considered low diet, excluding meat, fish, and poultry, would amount to no more than 3 per cent. At the General Hospital, Birmingham, the figures for one quarter show a daily average of 214 patients, of which an average of 27 were on milk diet (3 pints of milk a day), an average of 57 on low diet (bread, milk, and pudding), and about ·3 on middle diet, which includes broth and beef tea. The average number of patients altogether on these diets, which exclude meat, fish, or poultry, was 40 per cent.

It is clear from these figures, however fragmentary in the absence of further data they may appear, that, in comparing the cost of patients' food in one hospital with another, the proportion of those on low diet is an important point to gauge. T[illegible]ng them all together, they may be said to be boarded at no more than half the cost of the others, as will appear from a brief estimate of the cost of full diet.

Full diet consists of the following articles: An allowance of bread varying from 10 oz. to 1 lb., or, in the more modern hospitals, *ad lib.* The cost of bread is about 1d. per pound, and ¾d. a day represents the average consumption. Of cooked meat the common allowance is 6 oz. for male, and 4 oz. for female, patients. At Guy's, this allowance has been found to work out at an average consumed by each patient on meat diet of 8⅔ oz. of meat as received from the butcher, uncooked, with bone. The average cost of the joints given to the patients may be reckoned at 6d. per pound, so that 3d., or perhaps 3½d., a head must be assigned to this article of diet. Potatos, ½ lb., uncooked (at 9s. per hundredweight.), $\frac{1}{4}$d.; pudding, $\frac{3}{4}$d.; milk, 1 pint, 1$\frac{1}{4}$d.; soup or broth, 1 pint, 2d.; tea, sugar, butter, 1$\frac{1}{2}$d.; sundries, $\frac{1}{2}$d. The total is 10$\frac{1}{2}$d. This may be said to represent the minimum, but where tea, sugar, and butter are not provided the cost will be reduced to 9d. Fish is a common substitute for meat on one or two days in the week, and this might slightly reduce the cost. There are numberless small variations from this general standard. In some hospitals beer, in others a larger allowance of meat, increases the cost. And the continued fluctuation of prices according to locality, season, and management may easily upset a calculation dealing with such minute quantities.

In most hospitals the larger number of patients are on meat diet. At Guy's a calculation, made during 1890-91, showed a daily average of 265 on meat diet out of a total of 432 patients, or a proportion of 61 per cent. In the Birmingham General the proportion of those on meat diet for the quarter ending September 30th, 1895, was 129 out of a daily average of 214 patients, and this amounts to 60 per cent. At the Newcastle Royal Infirmary the percentage of patients on ordinary or meat diet for the last five years is 73.

Middle diet is usually a modification of full diet, consisting of slightly reduced quantities. Inquiry has shown that it is very little in use, and it need not, therefore, come under consideration.

But between low or fever diet and full diet lie a variety of menus for the patient known as special or extra diets. They consist usually of chicken, a quarter; rabbit, a quarter; chops, $\frac{1}{2}$ lb.; steak, $\frac{1}{2}$ lb.; eggs, jelly, arrowroot, beef tea, &c., and such expensive items as diabetic foods. (Wine and spirits are not included among provisions.) The cost of the diet may be easily doubled or trebled by extras. At Middlesex Hospital the cancer patients, who practically have what they like in the way of food, cost over three times as much as an ordinary patient. And at Guy's the paying patients in the Bright ward, for whom rather more variety is provided, cost 13s. 4d. per head per week, as against 5s. 4d. per head for the patients in the free wards.

Here, again, as in the case of the proportion of those on low diet, a wide variation exist. At one hospital the cost of extras is a heavy item; at another they will be almost unknown, and the most experienced persons have reached entirely opposite conclusions with respect to their need. Although they may, however, be sometimes recklessly abused by youthful house surgeons, it may be safely affirmed that where these simple variations of diet are not in use, a good deal of discomfort, if not of actual hardship, must fall on a certain proportion of the patients.

As a rule few hospitals err on the side of extravagance in the patients' food. The number to be fed is so large that any relaxation of economy becomes at once a serious burden to the institution. The evidence indeed points all the other way. It is not always clear that when due deduction has been made from the total cost of provisions for the board of members of the medical, nursing, and household staff the amount left over for the board of the patients is quite adequate to provide them with liberal diet of good quality. The practice of dividing the total cost of provisions equally among patients and officials alike to show the weekly average of expenditure for each inmate of the institution may easily give rise to an erroneous impression as to the amount spent on boarding each patient, and much vague dissatisfaction and irritation at complaint would find its proper outlet if the accounts were presented separately and committees were furnished with a plain demonstration of the patients' needs and the cost of meeting them.

It is doubtful whether a less sum than 4s. a week should ever be reckoned for each patient's board, except under special circumstances of numerous gifts in kind or very cheap food supplies. This allows for 40 per cent. of patients on low diet at a cost of 4d. per diem, and 60 per cent. of patients on full diet at a cost of 9d. per diem, including all extras. Where the average expenditure is less than this on each patient some evidence should be forthcoming that no deficiency in quantity or quality is tolerated.

